# Effects of Differences in the Increment Parameter Controlling Sector Numbers on Volumetric-Modulated Arc-Based Radiosurgery Planning With the Monaco® System for Single Brain Metastases

**DOI:** 10.7759/cureus.77324

**Published:** 2025-01-12

**Authors:** Kazuhiro Ohtakara, Kojiro Suzuki

**Affiliations:** 1 Department of Radiation Oncology, Kainan Hospital Aichi Prefectural Welfare Federation of Agricultural Cooperatives, Yatomi, JPN; 2 Department of Radiology, Aichi Medical University Hospital, Nagakute, JPN

**Keywords:** brain metastases, dose conformity, dose distribution, dose gradient, increment parameter, planning study, plan quality, stereotactic radiosurgery, volumetric-modulated arc therapy, x-ray voxel monte carlo

## Abstract

Purpose

A volumetric-modulated arcs (VMAs) technique, including non-coplanar arcs, enables efficient generation and implementation of suitable dose distribution in linac-based stereotactic radiosurgery (SRS) using a multileaf collimator (MLC) for brain metastases (BMs). In a Monaco^® ^treatment planning system (TPS) (Elekta AB, Stockholm, Sweden), an increment (Inc) parameter controls the number of sectors for each arc in VMA optimization. However, the optimal Inc parameter setting has remained to be determined. This study, therefore, aimed to investigate the impacts of differences in the Inc parameter on VMA-based SRS planning for BMs.

Materials and methods

This planning study targeted 30 clinical BMs with a gross tumor volume (GTV) of 0.08-48.09 cc (median 9.81 cc), which were previously analyzed. The treatment platform included a 5 mm leaf-width MLC Agility^®^ (Elekta AB, Stockholm, Sweden) and Monaco^®^ TPS (Elekta AB). The prescribed dose was uniformly assigned to the GTV near-minimum dose (DV-0.01 cc), the minimum dose of a GTV minus 0.01 cc, i.e., D>95% for GTV >0.20 cc and D95% for GTV ≤0.20 cc, to minimize the uncovered GTV to the equivalent of a 3 mm diameter lesion. The VMA planning was optimized to prioritize GTV dose conformity and the steepness of the dose gradient outside the GTV, without dose constraints within the GTV boundary, according to previously established methods. The Inc parameter settings of 10º, 20º, and 30º (Inc 10, Inc 20, and Inc 30) were compared, and all other parameters were unified.

Results

The Inc 30 was significantly inferior in GTV dose conformity and the steepness of dose gradients both outside and inside the GTV boundary, including the gradual dose attenuation margin outside the GTV surface. The Inc 10 had the most inhomogeneous GTV dose with the highest dose increase (2-4 mm) inside the GTV boundary and the longest total calculation time (tCT). The Inc 20 had the shortest tCT and showed a tendency to be superior in the concentric lamellarity of dose gradients 2 mm outside and 2-4 mm inside the GTV boundary. There was no significant difference between the Inc 10 and Inc 20, except for the superiority of the Inc 20 in the tCT and the concentric lamellarity of dose gradients.

Conclusions

The Inc parameter setting has significant impacts on the process and quality of treatment planning for VMA-based SRS for BMs. The Inc parameter of 20º per arc is recommended for templating in terms of the overall plan quality and the reasonable tCT. The Inc 10º can be an option if it is necessary to further enhance the steepness of dose increase inside the GTV boundary, although the tCT increases considerably.

## Introduction

With the increase in the number of patients harboring brain metastases (BMs) and advances in systemic therapy, the role of stereotactic radiosurgery (SRS) is increasing as an efficacious and least invasive local therapeutic avenue [1,2]. Implementing appropriate SRS using the most popular general-purpose Linac device is an increasingly important issue in providing treatment closer to more patients with BMs. Dynamic conformal arcs (DCA) are conventionally used for linac-based SRS using a multileaf collimator (MLC) [3,4]. Volumetric-modulated arcs (VMAs) are actively used for SRS and are particularly effective in irradiating irregularly shaped lesions and multiple lesions simultaneously [4,5]. VMAs are essential to achieving a suitable dose distribution, especially with a 5mm leaf-width MLC [6].

It is desirable to start the irradiation early after image acquisition to ensure treatment accuracy for particularly large lesions with massive surrounding edema, minimizing tumor growth and displacement during the waiting period [7]. VMA requires more complicated treatment planning, verification, and irradiation than DCA, including appropriate settings of various parameters relevant to treatment planning to implement effective and efficient irradiation [3,5,6]. A Monaco® (Elekta AB, Stockholm, Sweden) treatment planning system (TPS) supports VMA using a variety of cost functions (CFs) for optimization and an X-ray voxel Monte Carlo (XVMC) algorithm with high dose calculation accuracy [6,8]. We have previously investigated the optimal arc arrangement, CF selection and settings, calculation grid size, and statistical uncertainty of the XVMC algorithm for VMA-based SRS using Monaco^®^ [9-12]. We have also clarified the issues inherent in conventional dose prescription and evaluation metrics for general intracranial SRS, including dose prescription with specific percentage coverage of the gross tumor volume (GTV) with or without an isotropic margin, plan evaluation with conformity and gradient indices, and proposed alternatives that are more relevant to anti-tumor efficacy and toxicity [13-17]. Further efforts are needed to pursue the optimal irradiation method to maximize the functionality of the (existing) equipment and further improve treatment efficacy and safety.

Monaco^®^ requires the setting of an increment (Inc) mechanical limiting parameter that controls the number of generated sectors for each arc in VMA planning [18-22]. Very few studies have examined the Inc parameter values of 10-40º for VMA in the head and neck, chest, and pelvic regions [18-22]. However, the optimal Inc parameter value for SRS of intracranial small lesions has remained to be investigated. This study was conducted to examine the impact of differences in the Inc parameter setting on VMA-based SRS planning for single BMs and to determine the optimal value for a template for effective and efficient treatment planning after image acquisition.

This study was approved by the Clinical Research Review Board of Kainan Hospital Aichi Prefectural Welfare Federation of Agricultural Cooperatives (20240830-01).

## Materials and methods

The study population for this planning study included 30 lesions in 27 patients harboring BMs, for which multi-fraction SRS had been performed previously at our facility, to encompass a variety of sizes, shapes, and locations. The 30 lesions are the same as those examined in the previous studies [12]. Each of the 30 lesions was treated as a single BM. Each GTV was defined by multi-image co-registration using a dedicated software MIM Maestro^®^ version 7.1.3 (MIM Software Inc., Cleveland, OH, USA) as described previously [23,24]. The GTV ranged from 0.08 cc to 48.09 cc (median value: 9.81 cc; interquartile range (IQR): 4.38, 24.31 cc).

The treatment platform used was a 160-leaf, 5-mm leaf-width MLC Agility® (Elekta AB, Stockholm, Sweden) integrated into a linac Infinity® (Elekta AB, Stockholm, Sweden) with a flattening filter-free mode and a six MV X-ray beam, with a maximum dose rate of 1400 MU/min [25]. The TPS used was Monaco^®^ version 5.51.10 (Elekta AB, Stockholm, Sweden) [6,8].

VMA-based SRS planning for each GTV was uniformly performed according to the method determined to be optimal based on previous study results, except for the Inc parameter setting [10-12,15]. In this study, the Inc parameters of 10º, 20º, and 30º were compared. Each irradiation isocenter was set at the GTV center. The arc arrangement consisted of one coplanar arc with a 360º rotation and a collimator angle of 0º, and two non-coplanar arcs, each with a 180º rotation, collimator angles of 45º and 90º, and couch rotations of 60º clockwise and counterclockwise, respectively, dividing the cranial hemisphere into three equal parts [10]. The Pareto mode was adopted for VMA optimization. Three physical CFs were applied to each GTV and the patient’s head contour to maximize GTV dose conformity with the prescribed isodose surface (IDS) and the steepness of the dose gradient outside the GTV boundary, as previously described [12,15]. The same prescribed dose, e.g., 43.000 Gy in five fractions, was uniformly assigned to the GTV *D*_V-0.01 cc_, the minimum dose to cover a GTV minus 0.01 cc (*D*_>95%_ for GTV >0.20 cc and *D*_95%_ for GTV ≤0.20 cc), to minimize the uncovered tumor volume to the equivalent of ≤3 mm diameter lesion [15]. Consequently, the GTV coverage values by the *D*_V-0.01 cc_ ranged from 95.00% to 99.98% (median value: 99.90; IQR: 99.77, 99.96). In the settings of the sequence parameters for VMA, the Segment Shape Optimization option was included with high-precision leaf positions set to a value of 20, prioritizing planning quality. The maximum control points per arc were set to 1024, the minimum segment width to 0.5 cm, and medium fluence smoothing was applied. In the calculation properties, the grid spacing and statistical uncertainty of the XVMC algorithm were set to 0.1 cm and 1% per calculation, respectively, with dose deposition to the medium [12]. In the IMRT prescription parameters, the beamlet width was set to 0.30 cm, and both the target and avoidance margins were set to normal (8 mm), similar to previous studies. After the final dose calculation, each GTV coverage with the prescribed dose was rescaled according to the coverage value (≥95%) of GTV DV-0.01 cc [11,15]. A change in the GTV dose after rescaling was recorded to the third decimal place as a rescaling ratio, displayed as 'Dose rescaled by a ratio of X' on Monaco® [11,12]. The total calculation time (tCT) was defined as the time from optimization initiation to final control point completion and was determined from the optimization console on Monaco® [11,12].

For dosimetric comparison, isotropic margins of 2 mm, -2 mm, and -4 mm were added to each GTV boundary using MIM Maestro^®^ to generate the GTV +2 mm, GTV -2 mm, and GTV -4 mm structures, respectively [15-17]. The GTV -2 mm and GTV -4mm were generated only for GTVs of ≥0.72 cc (28 lesions) and ≥2.20 cc (27 lesions), respectively, to ensure the minimum meaningful volume for evaluation [17]. An irradiated isodose volume (IIV) was defined as the total volume irradiated with more than a certain relevant dose, including the GTV [26,27]. The IIVs of 100%, 75%, and 50% of the GTV DV-0.01 cc (100%, 75%, and 50% PIV, prescribed isodose volume) were calculated from the dose-volume histogram (DVH) for the volume generated by adding an isotropic margin of 10 mm to 30 mm to each GTV boundary CF. The IIVs may include tissues other than brain parenchyma, such as cerebrospinal fluid and bone. The absolute volumes obtained by subtracting the GTV from these IIVs were recorded as each spillage volume. The GTV near-maximum dose (Dnear-max) was recorded as the D0.01 cc, the minimum dose covering 0.01 cc of the GTV, for GTV ≥0.20 cc, or as D5% (D <0.01 cc) for GTV <0.20 cc [11,15]. The GTV dose inhomogeneity was recorded as the GTV DV-0.01 cc (%) relative to the GTV Dnear-max (100%) [11]. The near-minimum doses of the GTV, GTV +2 mm, GTV -2 mm, and GTV -4 mm structures were evaluated as each D_eIIV_ (eIIV: equivalent IIV), the minimum dose to cover an IIV equivalent to each reference target volume (TV) on the DVH, with a variable coverage value [16,17]. Each D_eIIV_ was recorded as a relative dose (%) to the GTV DV-0.01 cc (100%) [16,17]. The coverage value (%) of each reference TV by the D_eIIV_ was also recorded. While a high value of GTV D_eIIV_ indicates the steepness of the dose increase just inside the prescribed IDS, the closer the GTV D_eIIV_ is to the prescribed dose (GTV DV-0.01 cc), the better the dose conformity [17]. The GTV dose conformity was compared using the smallness of the 100% PIV spillage volume (cc), the high GTV coverage value (%) by the D_eIIV_, and the closeness of GTV D_eIIV_ to the GTV DV-0.01 cc. The appropriateness of the dose attenuation margin outside the GTV, i.e., the steepness and concentric lamellarity, was compared using the low GTV +2 mm D_eIIV_ (%) relative to the GTV DV-0.01 cc (100%) and the high GTV coverage value by the D_eIIV_ [16]. The steepness of the dose gradient outside the GTV was compared using the smallness of the 75% and 50% PIV spillage volumes [11,12]. The steepness and concentric lamellarity of the dose increase inside the GTV boundary were compared using the D_eIIV_s (%) of the GTV, GTV -2 mm, and GTV -4 mm along with each coverage value [11,16].

For statistical analyses, paired nonparametric tests were adopted, considering the dominant distributions of the variables. Box-and-whisker plots (BWPs) were used to represent the distributions of variables. In the BWP, the whiskers show the nearest values ≤1.5 times the IQR. The cross marks beyond the lines indicate the individual outliers >1.5 times the IQR. Friedman’s test (FT) and Scheffe’s post-hoc test (SPHT) were used to compare three numerical variables with correspondence. If there was no significant difference between the two numerical variables in the SPHT and the P-value was <0.9, the Wilcoxon signed-rank test (WSRT) was additionally applied to compare them. Jonckheere-Terpstra (JT) test was used to assess trends of increase or decrease in dosimetric parameters with the increase of Inc parameter between three variables. Statistical significance was considered at P<0.05 (*), P<0.01 (**), and P<0.001 (***). Statistical analyses were performed using BellCurve for Excel (version 4.05; Social Survey Research Information Co., Ltd., Tokyo, Japan).

## Results

The results of the planning and dosimetric comparisons between the three groups with the Inc parameters of 10º, 20º, and 30º (Inc 10, Inc 20, and Inc 30) are shown in Tables 1 and 2.

**Table 1:** Comparison between three planning with different Inc parameters: part 1. If a P-value of SPHT is <0.9, the result of WSRT is attached. Statistical significance is displayed in three levels: *P<0.05, **P<0.01, and ***P<0.001. Inc: increment; tCT: total calculation time; PIV: prescribed isodose volume; GTV: gross tumor volume; D_eIIV_: the minimum dose to cover the IIV equivalent to a reference TV; GTV +2 mm: GTV evenly expanded by 2 mm; X% PIV: the volume irradiated with ≥X% of the prescribed dose, including a TV; NS: not significant; IIV: irradiated isodose volume; TV: target volume; FT: Friedman’s test; SPHT: Scheffe’s post-hoc test; WSRT: Wilcoxon signed-rank test

Parameter	Inc	tCT (min)	Rescaling ratio	PIV spillage (cc)	GTV D_eIIV_ coverage (%)	GTV +2 mm D_eIIV_ (%)	GTV +2 mm D_eIIV_ coverage (%)	75% PIV spillage (cc)	50% PIV spillage (cc)
FT (P-value)		<0.001***	0.729 (NS)	0.061 (NS)	0.069 (NS)	<0.001***	0.327 (NS)	<0.001***	0.012*
SPHT (WSRT): P-value	10º vs. 20º	<0.001***	0.924 (NS)	0.998 (NS)	0.873 (NS) (0.206 (NS))	1.0000 (NS)	0.701 (NS) (0.530 (NS))	0.928 (NS)	0.812 (NS) (0.517 (NS))
	10º vs. 30º	0.036*	0.924 (NS)	0.114 (NS) (0.029*)	0.239 (NS) (0.175 (NS))	<0.001***	0.811 (NS) (0.627 (NS))	<0.001***	0.090 (NS) (0.0495*)
	20º vs. 30º	0.090 (NS) (0.055 (NS))	0.729 (NS) (0.135 (NS))	0.131 (NS) (0.063 (NS))	0.086 (NS) (0.021*)	<0.001***	0.329 (NS) (0.266 (NS))	<0.001***	0.018*

**Table 2:** Comparison between three planning with different Inc parameters: part 2. If a P-value of SPHT is <0.9, the result of WSRT is attached. Statistical significance is displayed in three levels: *P<0.05, **P<0.01, and ***P<0.001. Inc: increment; GTV: gross tumor volume; D_V-0.01 cc_: the minimum dose to cover a TV minus 0.01 cc; IDS: isodose surface; D_eIIV_: the minimum dose to cover the IIV equivalent to a reference TV; GTV - X mm: GTV evenly reduced by X mm; NS: not significant; IIV: irradiated isodose volume; TV: target volume; FT, Friedman’s test; SPHT: Scheffe’s post-hoc test; WSRT: Wilcoxon signed-rank test

Parameter	Inc	GTV D_eIIV_ %IDS (%)	GTV D_eIIV_ (%)	GTV -2 mm D_eIIV_ (%)	GTV -2 mm D_eIIV_ coverage (%)	GTV -4 mm D_eIIV_ (%)	GTV -4 mm D_eIIV_ coverage (%)
FT (P-value)		<0.001***	0.967 (NS)	<0.001***	0.0498*	<0.001***	0.021*
SPHT (WSRT): P-value	10º vs. 20º	0.010**	0.992 (NS)	0.134 (NS) (0.006**)	0.086 (NS) (0.016*)	0.0498*	0.891 (NS) (0.939 (NS))
	10º vs. 30º	<0.001***	0.967 (NS)	<0.001***	0.980 (NS)	<0.001***	0.033*
	20º vs. 30º	0.017*	0.992 (NS)	0.006**	0.132 (NS) (0.285 (NS))	0.001**	0.104 (NS) (0.014*)

**Figure 1:**
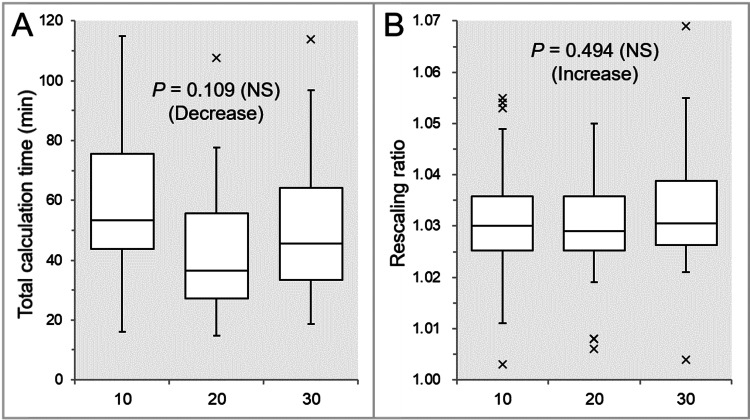
Comparison of tCT and rescaling ratio to align the prescription dose after optimization. The images show BWPs (A,B) for comparisons of the tCT (A) and rescaling ratio (B) between the three groups, respectively. The results of the JT test are attached along with the trends (A,B). NS: not significant; tCT: total calculation time; BWPs, box-and-whisker plots; JT, Jonckheere-Terpstra

The tCT was significantly longer in the Inc 10 than in the Inc 20 and Inc 30, while there was a trend of decrease in the Inc 20 than in the Inc 30 (Table 1 and Figure 1A).

There was no significant difference in the rescaling ratios between the three groups (Table 1 and Figure 1B), while the Inc 20 tended to have the smallest dispersion, and its median value was closest to one (Figure 1B).

**Figure 2:**
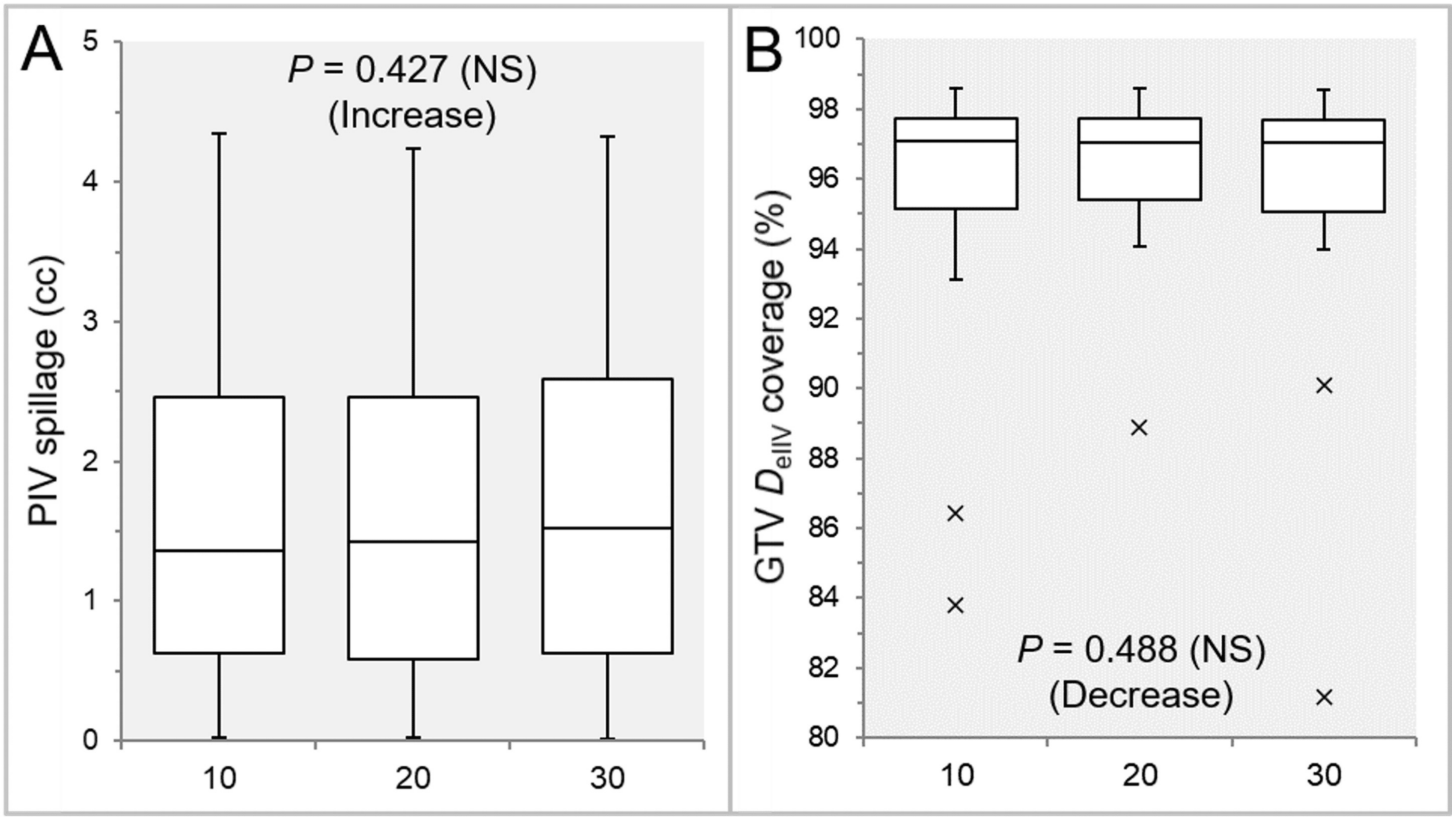
Comparison of GTV dose conformity. The images show BWPs (A,B), along with the results of the JT test, for comparisons of the PIV spillage volume outside the GTV boundary (A) and the GTV coverage value by the D_eIIV_ (B) between the three groups. GTV: gross tumor volume; PIV: prescribed isodose volume; D_eIIV_: the minimum dose to cover the IIV equivalent to a reference TV; NS: not significant; BWPs: box-and-whisker plots; JT: Jonckheere-Terpstra; IIV: irradiated isodose volume; TV: target volume

The PIV spillage was significantly smaller in the Inc 10 than in the Inc 30, and the PIV spillage in the Inc 20 tended to be smaller than in the Inc 30 (Table 1 and Figure 2A).

However, there was no significant difference between the Inc 10 and Inc 20 (Table 1 and Figure 2A). The GTV coverage value with the D_eIIV_ was significantly higher in the Inc 20 than in the Inc 30, while there was no significant difference between the Inc 10 and Inc 20 (Table 1 and Figure 2B).

**Figure 3:**
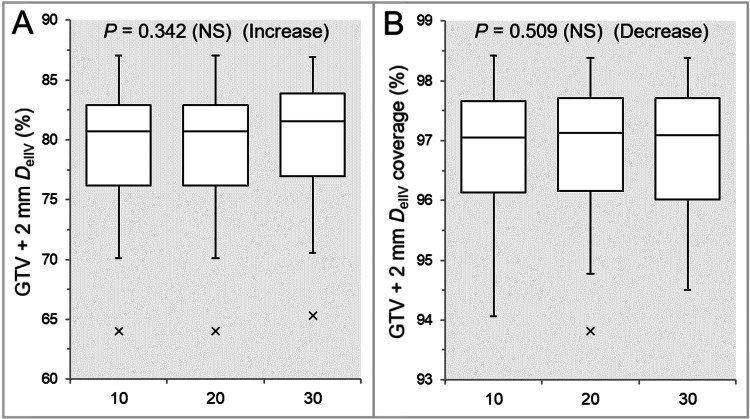
Comparison of the appropriateness of dose attenuation margin outside the GTV. The images show BWPs (A,B), along with the results of the JT test, for comparisons of the GTV +2 mm D_eIIV_ (%) relative to the GTV D_V-0.01 cc_ (100%) (A) and the coverage value of GTV +2 mm by the D_eIIV_ (B) between the three groups. GTV: gross tumor volume; GTV +2 mm: GTV evenly expanded by 2 mm; D_eIIV_: the minimum dose to cover the IIV equivalent to a reference TV; NS: not significant; BWPs: box-and-whisker plots; JT: Jonckheere-Terpstra; D_V-0.01 cc_: the minimum dose to cover a TV minus 0.01 cc; IIV: irradiated isodose volume; TV: target volume

The GTV +2 mm D_eIIV_ was significantly higher in the Inc 30 than in the Inc 10 and Inc 30, while there was no significant difference between the Inc 10 and Inc 20 (Table 1 and Figure 3A).

There was no significant difference in the coverage value of GTV +2 mm with the D_eIIV_, while that of the Inc 20 showed the highest tendency except for the outlier (Table 1 and Figure 3B).

**Figure 4:**
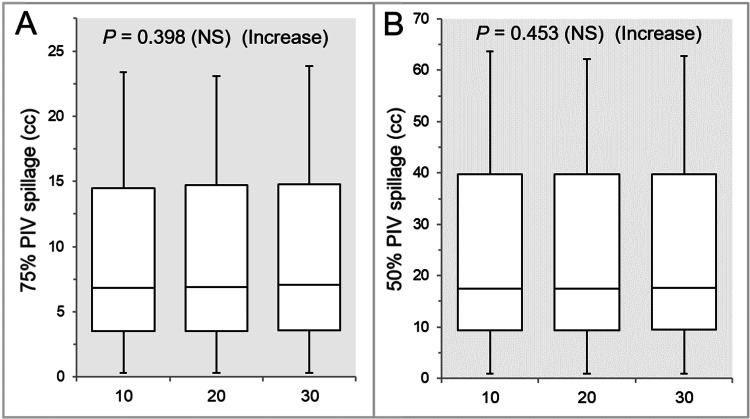
Comparisons of the steepness of dose gradient outside the GTV. The images show BWPs (A,B), along with the results of the JT test, for comparisons of the 75% (A) and 50% (B) PIV spillage volumes outside the GTV between the three groups. GTV: gross tumor volume; PIV: prescribed isodose volume; X% PIV: the isodose volume irradiated with ≥X% of the prescribed dose (GTV D_V-0.01 cc_), including a TV; D_V-0.01 cc_: the minimum dose to cover a TV minus 0.01 cc; NS: not significant; BWPs: box-and-whisker plots; JT: Jonckheere-Terpstra; TV: target volume

The 75% and 50% PIV spillages were significantly larger in the Inc 30 than in the Inc 10 and Inc 20, while there were no significant differences between the Inc 10 and Inc 20 (Table 1 and Figures 4A, 4B).

**Figure 5:**
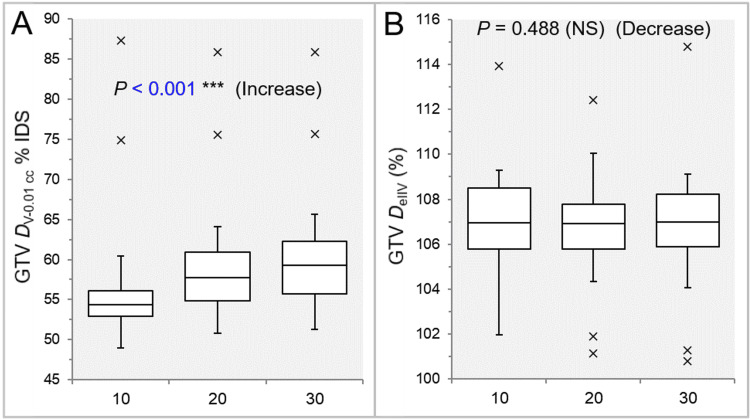
Comparisons of the GTV dose inhomogeneity and the steepness of dose increase just inside the prescribed IDS. The images show BWPs (A,B), along with the results of the JT test, for comparisons of the GTV D_V-0.01 cc_ (%) relative to the GTV near-maximum dose (D_0.01 cc_, 100%) (A) and the GTV D_eIIV_ (%) relative to the D_V-0.01 cc_ (100%) (B) between the three groups. GTV: gross tumor volume; D_V-0.01 cc_: the minimum dose to cover a TV minus 0.01 cc; IDS: isodose surface; D_eIIV_: the minimum dose to cover the IIV equivalent to a reference TV; NS: not significant; BWPs: box-and-whisker plots; JT: Jonckheere-Terpstra; D_0.01 cc_: the minimum dose covering 0.01 cc of a TV; IIV: irradiated isodose volume; TV: target volume

There was a significant trend of decrease in the GTV dose inhomogeneity, and the Inc 10 had the lowest GTV D_V-0.01 cc_ relative to the GTV D_near-max_ (Table 2 and Figure 5A).

**Figure 6:**
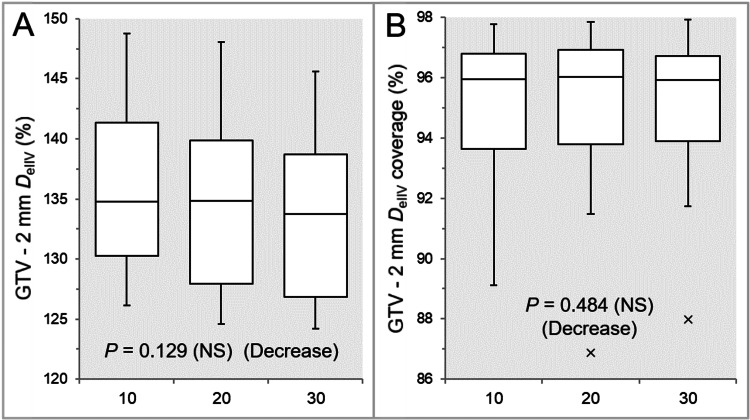
Comparison of the steepness of dose increase and the concentric lamellarity at 2 mm inside the GTV boundary. The images show BWPs (A,B), along with the results of the JT test, for comparisons of the GTV -2 mm D_eIIV_ (%) relative to the GTV D_V-0.01 cc_ (100%) (A) and the coverage value of GTV -2 mm by the D_eIIV_ (B) between the three groups. GTV: gross tumor volume; GTV -2 mm: GTV evenly reduced by 2 mm; D_eIIV_: the minimum dose to cover the IIV equivalent to a reference TV; NS: not significant; BWPs: box-and-whisker plots; JT: Jonckheere-Terpstra; D_V-0.01 cc_: the minimum dose to cover a TV minus 0.01 cc; IIV: irradiated isodose volume; TV: target volume

There was no significant difference in the GTV coverage value with the D_eIIV_ (Table 2 and Figure 5B). There was a trend of decrease in the GTV -2 mm D_eIIV_, and the Inc 10 had the highest value (Table 2 and Figure 6A).

**Figure 7:**
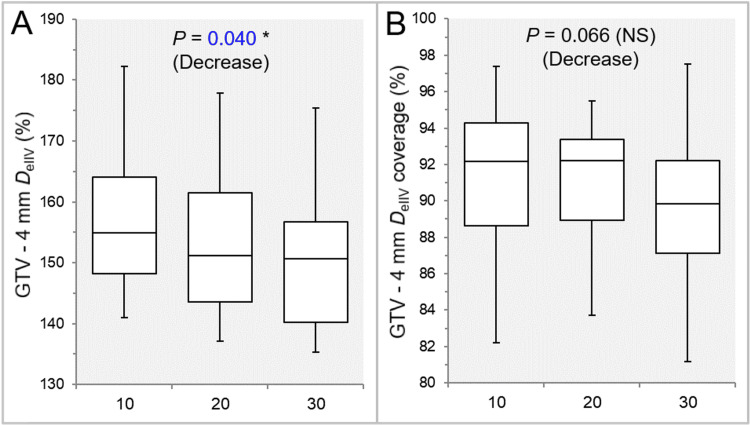
Comparison of the steepness of dose increase and the concentric lamellarity at 4 mm inside the GTV boundary. The images show BWPs (A,B), along with the results of the JT test, for comparisons of the GTV -4 mm D_eIIV_ (%) relative to the GTV D_V-0.01 cc_ (100%) (A) and the coverage value of GTV -4 mm by the D_eIIV_ (B) between the three groups. GTV: gross tumor volume; GTV -4 mm: GTV evenly reduced by 4 mm; D_eIIV_: the minimum dose to cover the IIV equivalent to a reference TV; NS: not significant; BWPs: box-and-whisker plots; JT: Jonckheere-Terpstra; D_V-0.01 cc_: the minimum dose to cover a reference TV minus 0.01 cc; IIV: irradiated isodose volume; TV: target volume

The coverage value of GTV -2 mm with D_eIIV_ was significantly higher in the Inc 20 than in the Inc 10, while there was no significant difference between the Inc 10 and Inc 30 or between the Inc 20 and Inc 30 (Table 2 and Figure 6B). There was a significant trend of decrease in the GTV -4 mm D_eIIV_, and the Inc 10 had the highest value (Table 2 and Figure 7A).

There was a trend of decrease in the coverage value of GTV -4 mm with the D_eIIV_, and the Inc 30 showed the lowest value (Table 2 and Figure 7B). The Inc 20 showed the lowest dispersion and the highest median value and first quartile (Table 2 and Figure 7B).

## Discussion

The appropriate Inc parameter setting is indispensable for DCA planning using Monaco^®^ [28,29]. Monaco^®^ also supports DCA based on inverse planning as DCAT (dynamic conformal arc therapy) with variable gantry rotation speed and dose rate [28,29]. The control points are placed at regular intervals based on half of the Inc parameter setting of each arc, i.e., control points every 10º of arc rotation at the Inc setting of 20º. However, we were careless about the Inc parameter setting in general VMA, including VMA-based SRS for BMs, and sometimes chose Inc of 30º, before conducting this study.

This planning study revealed that the differences in the Inc parameter setting between 10º and 30º have significant impacts on the plan quality and the tCT in VMA-based SRS for BMs. The Inc 20º had the shortest tCT and the smallest rescaling of the prescribed dose, while the Inc 30º had the worst steepness of dose falloff outside the GTV, and was disadvantageous in terms of reducing surrounding brain dose. The Inc 10º had the most inhomogeneous GTV dose with the steepest dose increase inside the GTV boundary, while there was no significant difference in normal tissue sparing between the Inc 10º and Inc 20º. The Inc 10º had the longest tCT.

Previous limited studies indicated that the differences in the Inc parameter, 10-40º, have significant impacts on the plan quality of VMA for cancer of various organs [18-22]. However, the optimal Inc parameters vary depending on the report and lesion site, and smaller is not necessarily better [18,22]. Taken together, the Inc parameter of 20º per arc is recommended as a template for VMA-based SRS using Monaco^®^, considering the quality and efficiency of treatment planning comprehensively. The Inc 10º can be an option if it is necessary to further enhance the steepness of dose increase inside the GTV boundary [30], although the tCT increases considerably. 

For effective and efficient VMA planning, it is necessary to appropriately set various parameters for optimization other than Inc in Monaco® TPS. It is important to recognize that the efficiency and quality of the treatment plan vary greatly depending on the settings, even for just Inc. In the future, it would be ideal if VMA-based SRS planning would be automatically optimized with a reasonable plan and irradiation time without the need to set Inc.

Study limitation

In this study, the Inc parameter settings other than 10, 20, and 30º, i.e., 15º and 40º, were not examined. In addition, a combination of different Inc parameters in the dual arc was not adopted due to the simple arc arrangement of three single arcs [21]. Whether the Inc 15º is better overall than 20º requires comparison, including the difference in the tCT. This study was also limited to SRS for single BMs. It is necessary to verify whether Inc 20º is optimal for simultaneous irradiation of multiple lesions, including differences depending on the number of lesions. This study is also limited to comparing treatment plans on a TPS, and the superiority of Inc 20º should be determined comprehensively, including the patient's specific quality assurance, delivery time, and clinical outcomes.

## Conclusions

Differences in the Inc parameter setting have significant impacts on the efficiency and quality of treatment planning for VMA-based SRS for BMs using Monaco^®^ TPS. The Inc parameter of 20º per arc is recommended as a template for effective and efficient planning after image acquisition in terms of the overall plan quality and reasonable tCT. The Inc 10º can be an option if it is necessary to further enhance the steepness of dose increase inside the GTV boundary, although the tCT increases considerably.
